# Renal cancer-selective Englerin A induces multiple mechanisms of cell death and autophagy

**DOI:** 10.1186/1756-9966-32-57

**Published:** 2013-08-20

**Authors:** Richard T Williams, Alice L Yu, Mitchell B Diccianni, Emmanuel A Theodorakis, Ayse Batova

**Affiliations:** 1 Department of Pediatrics, Hematology/Oncology, University of California, San Diego, CA, USA; 2 The Genomics Research Center, Academia Sinica, University of California, Taipei, Taiwan; 3 Stem Cell & Translational Cancer Research Center, Chang Gung Memorial Hospital, Linkou, Taoyuan, Taiwan; 4 Department of Chemistry and Biochemistry, University of California, 9500 Gilman Drive, La Jolla, CA 92093, USA

**Keywords:** Englerin A, Apoptosis, Autophagy, Necrosis, Renal cell carcinoma

## Abstract

Renal cell carcinoma (RCC), the most common malignancy of the kidney, is refractory to standard therapy and has an incidence that continues to rise. Screening of plant extracts in search of new agents to treat RCC resulted in the discovery of englerin A (EA), a natural product exhibiting potent selective cytotoxicity against renal cancer cells. Despite the establishment of synthetic routes to the synthesis of EA, very little is known about its mechanism of action. The results of the current study demonstrate for the first time that EA induces apoptosis in A498 renal cancer cells in addition to necrosis. The induction of apoptosis by EA required at least 24 h and was caspase independent. In addition, EA induced increased levels of autophagic vesicles in A498 cells which could be inhibited by nonessential amino acids (NEAA), known inhibitors of autophagy. Interestingly, inhibition of autophagy by NEAA did not diminish cell death suggesting that autophagy is not a cell death mechanism and likely represents a cell survival mechanism which ultimately fails. Apart from cell death, our results demonstrated that cells treated with EA accumulated in the G_2_ phase of the cell cycle indicating a block in G_2_/M transition. Moreover, our results determined that EA inhibited the activation of both AKT and ERK, kinases which are activated in cancer and implicated in unrestricted cell proliferation and induction of autophagy. The phosphorylation status of the cellular energy sensor, AMPK, appeared unaffected by EA. The high renal cancer selectivity of EA combined with its ability to induce multiple mechanisms of cell death while inhibiting pathways driving cell proliferation, suggest that EA is a highly unique agent with great potential as a therapeutic lead for the treatment of RCC.

## Introduction

Renal cell carcinoma (RCC) is the most common type of malignant kidney tumor with an incidence that continues to rise. Between 1992 and 2005, the incidence of RCC rose by 1.8% and 2.1% among white men and white women, respectively [1]. Although surgery can be curative for tumors confined to the kidney, about 25% of patients have metastatic disease at diagnosis, and another 20-40% develop metastases following surgery [2,3]. The two-year survival rate for patients with metastatic disease is under 20% due to the poor response of these tumors to current treatments. Clear cell RCC (cc-RCC) which comprises 83% of RCC is one of the most radio- and chemo-resistant cancers and no curative treatment is available once metastases develop [4].

Investigations of the molecular biology of RCC have established that inactivating alterations in the Von Hippel Lindau (VHL) tumor suppressor gene are present in the majority (91%) of sporadic cc-RCC underscoring the central role of VHL in the regulation of growth and differentiation of renal epithelium [5-7]. The VHL gene product is involved in oxygen and energy sensing by regulating the activity of the hypoxia inducible factors (HIFs) [8]. Inactivation of VHL results in HIF stabilization and the activation of transcription of over 60 hypoxia-responsive genes involved in oncogenesis and tumor progression including vascular endothelial growth factor (VEGF), the platelet-derived growth factor (PDGF), transforming growth factor alpha (TGF-α), epidermal growth factor (EGF), and glucose transporter-1 (GLUT-1) among others [9,10]. Subsequent to the activation of HIF-inducible genes, a variety of downstream signaling pathways are activated of which the most studied are the RAF-MEK-ERK series of kinases and the phosphatidylinositol-3 kinase-protein kinase B-mammalian target of rapamycin (PI3K-AKT-mTOR) pathway [11]. Based on the activation of these pathways in RCC, several targeted therapies have been developed including those against VEGF and PDGF receptors, and mTOR. However, despite the promise of approved targeted therapies for RCC, a complete response is rare and patients often become resistant/refractory to first line treatment [3,12]. Thus, new agents with improved efficacy and decreased toxicity are needed as treatment options in first line or subsequent settings.

The need to identify new chemical motifs as potential drug leads has spurred the screening of plant extracts that are being used in traditional medicines [13,14]. In particular, South Africa has a remarkable botanical diversity with over 30,000 flowering species, from which more than 3,000 are used for medicinal purposes throughout the country. Among them, plants of the genus Phyllanthus (Euphorbiaceae) are widely distributed in tropical forests throughout the world and have long been used in folk medicine to treat kidney and urinary tract infections [15]. Based on this knowledge, Ratnayake et al. [16] at the NCI screened extracts of the Tanzanian plant Phyllanthus engleri and have reported the isolation of two novel bioactive sesquiterpenes, named englerin A (EA) and englerin B. Initial studies by the NCI demonstrated that EA possessed very potent growth inhibitory activity (GI_50_ = 10–87 nM) against most RCC with a selectivity that is approximately 1,000-fold higher compared to other cancers.

Although several synthetic routes toward the synthesis of EA have been established [16-21], other than EA’s selective toxicity to RCC, recently confirmed by us [21], very little is known about its biological actions and mechanism(s) of action. Only recently, one study reported that EA induced necrosis in RCC [22]. The most recent report concluded that EA bound and activated protein kinase C-θ (PKCθ) to inhibit insulin signaling while, concurrently, activating HSF1, a known inducer of glucose dependence [23]. This dual signaling, that promotes glucose addiction while inhibiting glucose uptake by the cells, was proposed to be the mechanism for the selective cytotoxicity of EA. Although the data presented is compelling, whether in fact this mechanism accounts for the cytotoxicity of EA is not yet clear. Based on its cytotoxicity profile against the NCI60 cell panel, EA is clearly a very unique agent and there is much to be learned about the actions of EA in RCC and the mechanisms and targets involved in these actions. In this study, using the highly EA-sensitive A498 human renal carcinoma cells as our model system, we report the results of a thorough and systematic investigation to uncover the mechanisms of growth inhibition and cell death induced by EA and reveal for the first time that EA induces multiple mechanisms of cell death as well as cell cycle arrest while inducing autophagy.

## Material and methods

### Cell lines

The A498 human kidney carcinoma cell line was purchased from ATCC and maintained in RPMI medium supplemented with 10% FBS and 100 units/ml penicillin/streptomycin (complete medium).

### Reagents

Englerin A was purchased from Cerilliant Corporation. (Round Rock, Texas). Rapamycin was purchased from Enzo Life Sciences (Farmingdale, NY) as part of the Cyto-ID^®^ Autophagy Detection Kit. VP16 was purchased from Sigma Aldrich (St. Louis, MO). MEM 100X non-essential amino acids (NEAA) was purchased from Gibco Life Technologies (Grand Island, NY). Antibody against caspase-3 was a gift from Dr. Robert Naviaux and anti LC3B was purchased from Cell Signaling Technology (Danvers, MA). Antibody against β-actin (AC-15) was purchased from Sigma Aldrich (St. Louis, MO). Antibodies against phospho AMPK (Thr172) and phospho ERK (Thr202/Tyr204) as well as those for AMPK and ERK were generous gifts of Dr. R. Naviaux. The antibodies against AKT and phospho AKT (Ser473) were purchased from Cell Signaling Technology.

### Viability assay

A498 cells were plated at 5,000 cells/well in a 96-well plate in complete medium. The following day, cells were treated with EA at 50 and 100 nM. Control cells received 0.1% DMSO. All conditions were performed in triplicate. Cells were then incubated with additions for 24 or 48 h before measuring viability using the PrestoBlue^®^ (Invitrogen, CA) assay as described by manufacturer. This assay uses a resazurin-based solution that functions as a cell viability indicator by using the reducing power of living cells to quantitatively measure the proliferation of cells. Viability was determined by measuring fluorescence on a Synergy Mx plate reader (BioTek Instruments Inc., Winooski, VT) with excitation/emission at 560/590 nM.

### Apoptosis assays

Apoptosis was determined independently by two different methods. The Alexa Fluor^®^ 488 annexin V/Dead Cell Apoptosis Kit (Life Technologies, Grand Island, NY) was used to measure externalized phosphatidyl serine and dead cells permeable to propidium iodide (PI). For these experiments, A498 cells were treated with 100 nM EA or with 0.1% DMSO (control) for 24 and 46 h. Cells were then trypsinized, washed with ice cold PBS, and stained with Alexa Fluor^®^ 488 annexin V and PI as recommended by manufacturer. Cells were then analyzed by flow cytometry using a FACS Caliber flow cytometer (Beckton Dickinson, Franklin Lakes, NJ) and Flow Jo software (TreeStar Inc., Ashland, OR).

Apoptosis induced by EA in A498 cells was also determined by measuring cytoplasmic histone-associated-DNA-fragments using the Cell Death Detection ELISAPLUS kit (Roche Diagnostics GmbH, Mannheim, Germany) according to the manufacturer’s instructions. In these experiments, A498 cells were plated at 5,000 cells/well (96-well plate) in complete RPMI medium. The following day, cells were treated with 100 nM EA or with 0.1% DMSO, and incubated at 37°C for 18, 24, and 45 h before apoptosis was measured.

### Caspase assays

Multiple caspases were analyzed using the FLICA reagent (FAM Caspase Activity kit, Imgenex, San Diego, CA) which only binds active caspases. In these experiments, A498 cells were plated at 0.5 × 10^6^ cells/T-25 flask in complete RPMI. After cells were allowed to attach overnight, cells were treated with 100 nM EA or 0.1% DMSO for 43 h, or with 200 μM etoposide for 24 h. Cells were then harvested and stained with the FLICA reagent according to manufacturer’s recommendations and fluorescence was measured with excitation at 490 nm and emission at 520 nm. Caspase-9 activity was measured after treatment of cells with and without 100 nM EA as above followed by trypsinization and cell lysis. Caspase-9 activity was then determined using the Caspase-9 Colorimetric Assay kit (BioVision, Mountain View, CA) according to protocol provided by manufacturer. Absorbance was read at 400 nm. The levels of active caspase-3 were determined by Western blot analysis as described below.

### Autophagy assays

Autophagy was determined by three different methods including flow cytometry, fluorescence microscopy and western blot analysis. For flow cytometry experiments, A498 cells were plated in T-75 flasks at 1.25 × 10^6^/flask in complete RPMI. After the cells were allowed to attach overnight, cell were treated with 200 nM EA or 0.1% DMSO (control) for 46 h and with 500 nM rapamycin for 20 h. Autophagy was measured by staining autolysosomes and earlier autophagic compartments with the fluorescent probe Cyto-ID^®^ Green (Enzo Life Sciences, Farmingdale, NY) as recommended by manufacturer. Samples were then analyzed in the green (FL1) channel of the FACS Caliber flow cytometer.

For fluorescence microscopy, A498 cells were plated in complete RPMI on coverslips placed in a 60 mm dish at 1.5 (control cells) to 3.0 × 10^5^ (treated cells) cells/dish. After the cells were allowed to attach overnight, cell were treated with 200 nM EA or 0.1% DMSO (control) for 45 h. Cells were then stained with Hoechst nuclear stain and Cyto-ID^®^ Green detection reagent using the Cyto-ID^®^ Autophagy Detection Kit according to recommendations. Cells were fixed with 4% formaldehyde for 20 min at room temp followed by three washes with 1X assay buffer. Cover slips were then placed on slides with mounting media. Stained cells were analyzed by fluorescence microscopy (Olympus BX51 microscope that has been equipped with the fluorescence illuminator BX-URA2) using an Omega Optical XF100-2 filter for green bandpass with a 475 nm exciter to image autophagic cells.

### Western blot analysis

A498 cells were plated at 1–2 × 10^6^ cells/ T-75 flask in complete RPMI. After cells were allowed to adhere overnight, cells were treated with 100, 200 nM EA or with 0.1% DMSO for 48 h before harvesting. Cells were trypsinized, collected, and resuspended in ice- cold PBS. Cells were lysed in RIPA buffer (50 mM Tris–HCl pH 8.0, 1% Triton X-100, 150 mM NaCl, 1mM EDTA, 0.5% Deoxycholate, 0.1% Sodium Dodecyl Sulfate, 1mM Sodium Fluoride, 1 mM Sodium Pyrophosphate) in the presence of PMSF and protease inhibitor cocktail. Lysates were clarified by centrifugation for 15 min at 10,000×g, 4°C. To the clarified lysate, 4 × NuPAGE LDS sample buffer (Life Technologies) and 0.05 M dithiothreitol were added and samples were heated for 10 min at 80°C. Proteins were separated by SDS-PAGE on a 10% Bis-Tris NuPAGE Gel (Life Technologies) and then transferred to PVDF membranes (Bio-Rad). The PVDF membranes were blocked with 5% Bovine Serum Albumin (Sigma) in TBS with 0.05% Tween-20 and probed with antibodies against caspase-3, (diluted 1:1000), LC3B (diluted 1:1000), and B-actin (diluted 1:50,000). Antibodies against AMPK, AKT, ERK and against the corresponding phospho proteins were each diluted 1:1000 except for phospho AKT which was diluted at 1:500. An HRP-conjugated anti-mouse antibody (diluted 1:1000) or HRP-conjugated anti-rabbit antibody (diluted 1:1000;) was used as a secondary detection probe. Bands were visualized using ECL enhanced chemiluminescent substrate (Pierce) and exposed to HyBlot CL film (Denville Scientific). The film was developed with a Kodak film developer.

### Cell cycle analysis

A498 cells were plated at 2 × 10^5^ (control) or at 4 × 10^5^ (EA treated) cells/flask into T-25 flasks in complete RPMI. After cells were allowed to attach overnight, cells were treated with 200 nM EA or with 0.1% DMSO for 45 h. The cells were then trypsinized, washed with ice-cold PBS, fixed with ice-cold 70% ethanol at a 1:10 ratio of cell suspension to 70% ethanol, and stored at -20ºC overnight. Cells were washed twice with PBS and then stained with staining solution containing Triton x-100 (0.1% v/v), DNase free RNase (200 μg/ml), PI (30 μg/ml) in PBS for 15 min at 37ºC. PI content of cells was measured using a FACS Calibur flow cytometer and cell cycle distribution was determined using FlowJo analysis software.

## Results

### Examination of viability and determination of apoptosis and necrosis

**Figure 1:**
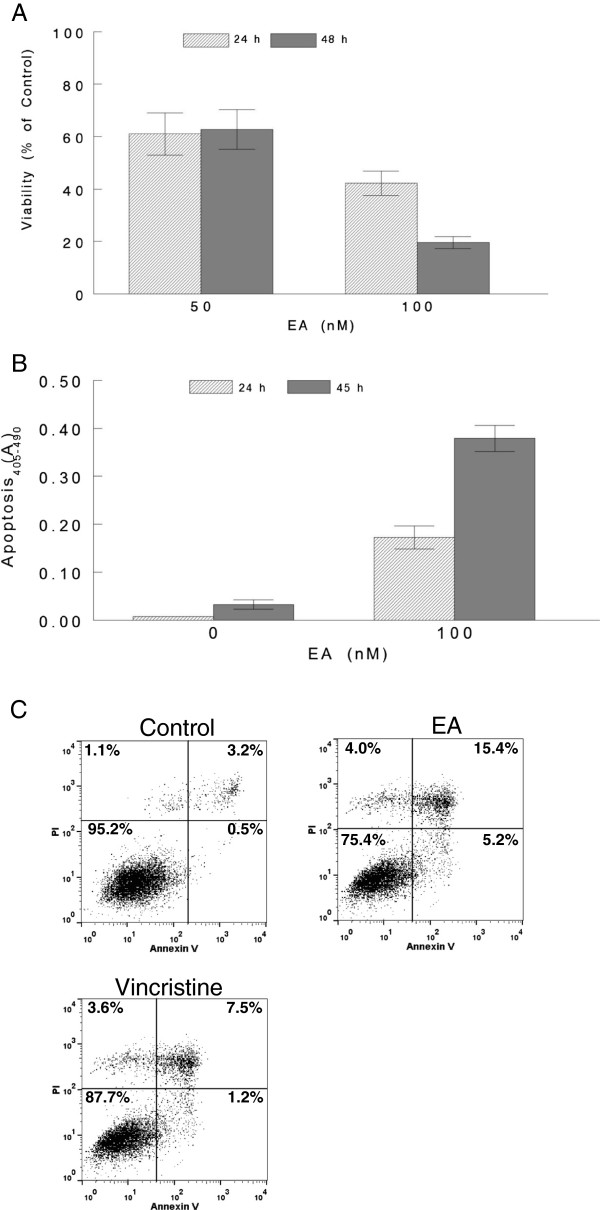
**Induction of cell death by EA in A498 RCC cells.** A498 cells were cells were treated with EA at 50 and 100 nM. Control cells received 0.1% DMSO (vehicle). All conditions were performed in triplicate. Cells were then incubated with additions for 24 or 48 h before measuring viability using the PrestoBlue^®^ assay **(A)**. A498 cells were treated with 100 nM EA or vehicle for 24 and 45 h durations. Apoptosis was determined by measuring cytoplasmic histone-associated-DNA-fragments using the Cell Death Detection ELISAPLUS assay kit **(B)**. A498 cells were treated with 100 nM EA or with 0.1% DMSO (control) for 24 and 46 h. Cells were then trypsinized, washed with ice cold PBS, and stained with Alexa Fluor^®^ 488 annexin V and PI and analyzed by flow cytometry **(C)**.

Examination of the cytotoxicity of EA against multiple tumor types using the NCI60 cell panel determined that EA was very selectively toxic to RCC with GI_50_ concentrations ranging from 10–83 nM in most RCC lines [16]. Our own previous studies have also documented this selectivity [21]. We extended these results by conducting viability studies using one of the most sensitive RCC lines, A498 cells, and treated them with 50 and 100 nM EA from 24 to 48 h. The results of these experiments which measured metabolically active cells, indicated that although cell death was observed by 24 h at both EA concentrations, the majority of cell death (> 80% of control) required greater than 24 h and occurred by 48 h of treatment (Figure 1A). To confirm these results, as well as to determine the cell death mechanism(s) involved in EA-induced cell death, apoptosis was determined by measuring histone-associated DNA fragments by ELISA in A498 cells treated with 100 nM EA for 24 and 45 h (Figure 1B). The induction of apoptosis by EA in A498 cells required at least 24 h for significant levels of apoptosis to occur as no apoptosis was observed at 18 h (data not shown). Additional studies determined that the EA-induced apoptosis was also dose-dependent (data not shown). To further confirm that EA induced apoptosis in A498 cells, apoptosis was also determined by measuring phosphatidylserine exposure on cells using the Alexa Fluor^®^ 488 annexin V/Dead Cell Apoptosis kit followed by flow cytometry. The results of these experiments revealed that EA at 100 nM induced apoptosis in A498 cells at levels well above control by 46 h of treatment (Figure 1C). The apoptotic cells included Annexin V positive (5.2%) as well as Annexin V/PI double positive (15.4%) cells representing early and late stages of apoptosis, respectively. In addition, some necrotic, PI positive, only (4.0%), cells were also observed. Furthermore, cells treated with a clinically relevant concentration (50 nM) of vincristine, a chemotherapeutic agent known to induce apoptosis in several tumor types [24], induced similar levels of necrosis (3.6%), but less than half as much apoptosis (1.2% and 7.5% early and late stages of apoptosis, respectively) as EA in A498 cells. Higher concentrations of vincristine were not tested, thus, it is possible that 100 nM vincristine may have induced similar levels of apoptosis to EA. Overall, our results indicated that EA induced cell death in A498 cells, the majority of which, occurred after 24 h of treatment, and at least part of this cell death was due to apoptosis.

### Analysis of caspase activity

**Figure 2:**
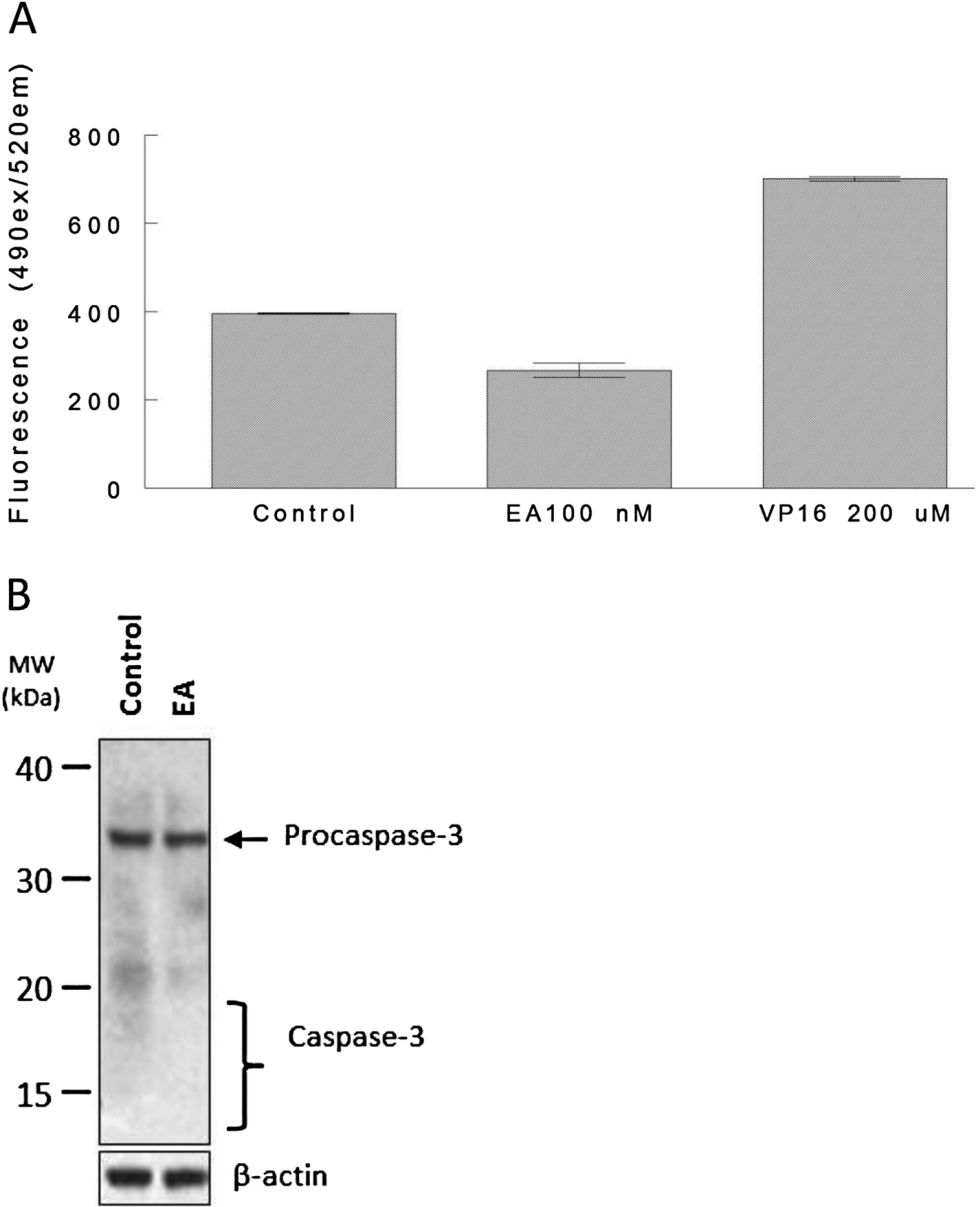
**Caspases are not activated in-EA induced cell death.** A498 cells cells were treated with 100 nM EA or 0.1% DMSO (control) for 43 h, or with 200 μM etoposide for 24 h. Cells were then harvested and stained with the FLICA reagent which only binds active caspases. Levels of active caspase were then determined by fluorescence **(A)**. A498 cells were treated with 200 nM EA or with 0.1% DMSO (control) for 48 h and protein was extracted. Western blot analysis was performed using an anti-caspase-3 antibody. B-actin was probed as a control for protein loading **(B)**.

Having established that EA induced apoptosis in A498 cells, the question remained as to whether caspases were involved in EA-induced apoptosis and if so which ones were involved. To determine if EA induced caspase activation in general, active caspases were measured in A498 cells, treated as indicated in Figure 2A, by using the FLICA reagent (Fluorochrome Inhibitor of Caspases) which binds covalently to only active caspases and allows active caspase detection by fluorescence. The etoposide, VP16, a chemotherapeutic agent known to induce apoptosis in multiple tumor types and known to activate caspases [25], was used as a positive control in these experiments. Because the effective dose of VP16 is in the micromolar range and since RCC cells are not nearly as sensitive to VP16 and other standard chemotherapeutic agents when compared to EA, higher concentrations of VP16 were used in these experiments over EA. While active caspases were detected in cells treated with 200 μM VP16, active caspases were not detected in cells treated with 100 nM EA (Figure 2A), a concentration of EA reducing cell viability by 70-80%. To confirm that EA did not induce caspase activation, levels of active caspase-3, an executioner caspase, were also determined. Levels of active caspase-3 were examined by Western Blot analysis in A498 cells treated with 200 nM EA or 0.1% DMSO for 48 h. The results of this analysis showed no evidence of caspase-3 activation by EA (Figure 2B) confirming our results using the FLICA reagent (Figure 2A). Similarly, active caspase-9, a caspase frequently activated by anti-cancer agents, was also not detected in A498 cells treated with EA (data not shown). Altogether, our results indicate that apoptosis induced by EA in A498 cells occurs in a caspase-independent manner.

### Detection of autophagy

**Figure 3:**
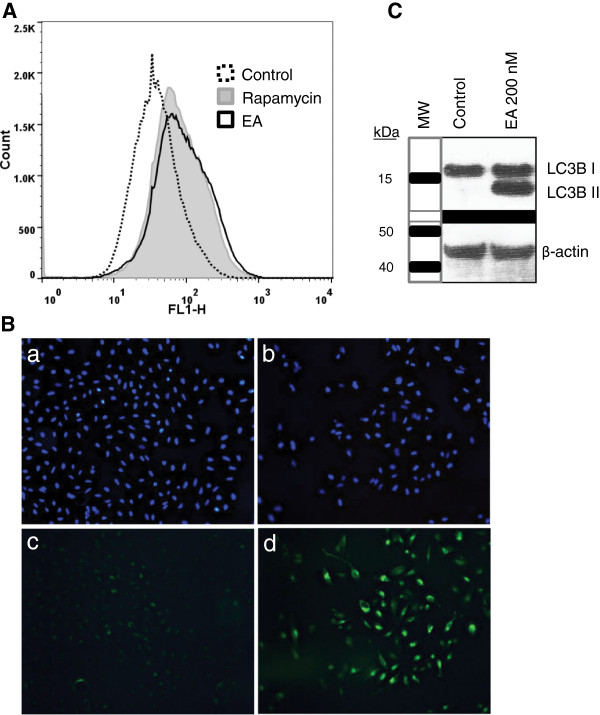
**EA induces autophagy in A498 cells.** A498 cells were treated with 200 nM EA or 0.1% DMSO (control) for 46 h and with 500 nM rapamycin for 20 h. Autophagy was measured by staining autolysosomes and earlier autophagic compartments with the fluorescent probe Cyto-ID^®^ Green. Samples were then analyzed in the green (FL1) channel of the FACS Caliber flow cytometer **(A)**. Cells were treated with 200 nM EA or 0.1% DMSO (control) for 45 h and then stained with Hoechst nuclear stain and Cyto-ID^®^ Green detection reagent followed by fixing with 4% formaldehyde. The stained cells were then analyzed by fluorescence microscopy. Panels a and c show cells treated with 0.1% DMSO and panels b and d show cells treated with EA. Nuclei are stained in blue. Autolysosomes and earlier autophagic compartments are stained in green **(B)**. A498 cells were treated with 200 nM EA or with 0.1% DMSO (control) for 48 h and protein was extracted. Western blot analysis was performed using an anti-LC3B antibody. B-actin was probed as a control for protein loading **(C)**.

**Figure 4:**
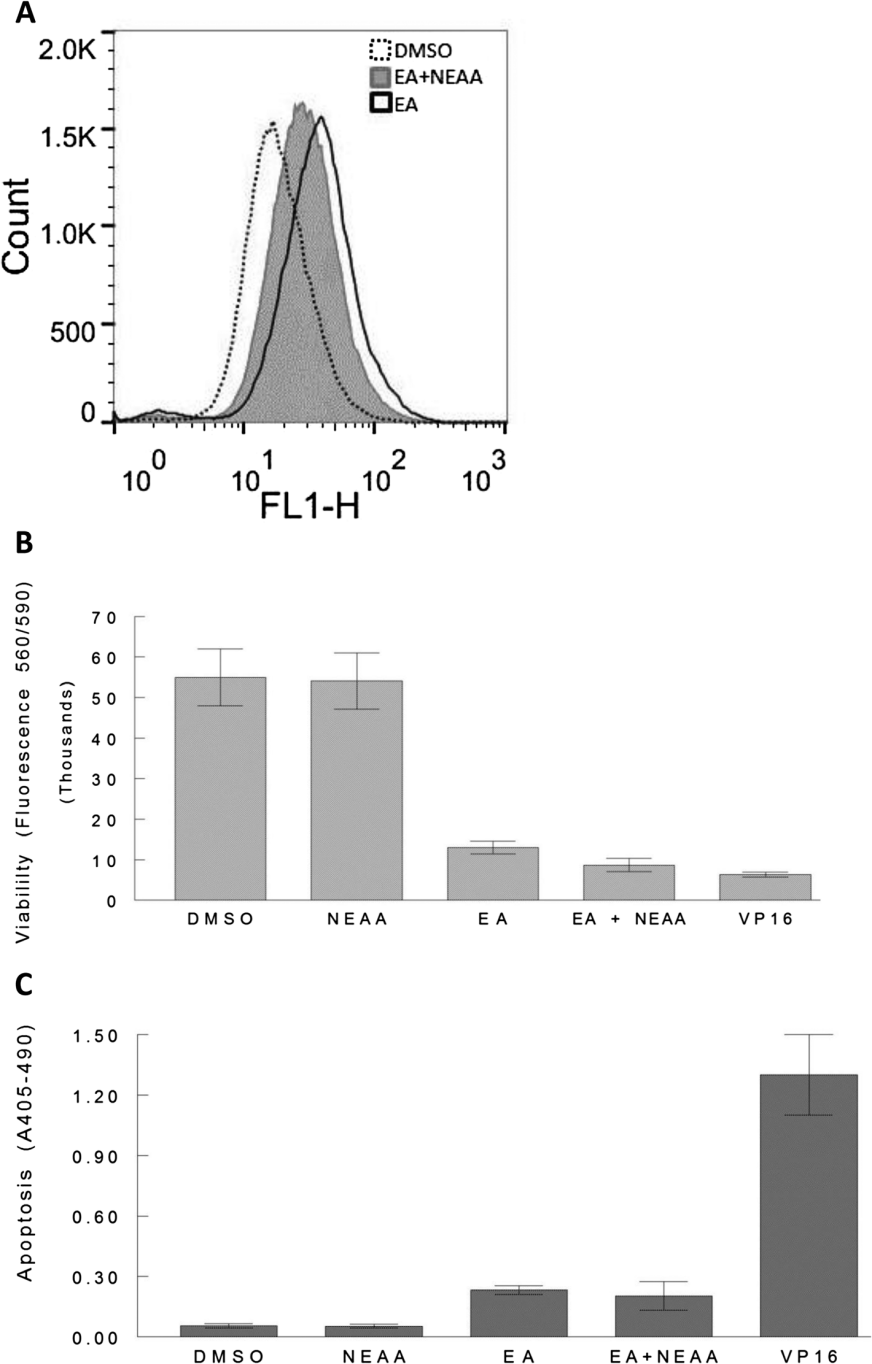
**Inhibition of autophagy does not affect EA-induced cell death.** A498 cells were cultured in the presence and absence of 100 nM EA or, with EA and 1X NEAA in combination for 44 h. Autophagy was then determined by flow cytometry after staining with Cyto-ID^®^ **(A)**. A498 cells were treated with 150 nM EA, 0.1% DMSO, 1X NEAA, 200 μM VP16 or with 100 nM EA plus 1X NEAA for 46 h. Cell viability was then determined using the PrestoBlue^®^ assay **(B)**. A498 cells were treated as in **(B)** and then apoptosis was determined by measuring histone-associated DNA fragments by ELISA **(C)**.

The finding that apoptosis induced by EA in A498 cells required at least 24 h, even at concentrations above the LC_50_ of 75 nM (16), is in contrast to many chemotherapeutic agents such as camptothecin and doxorubicin that require less than 8 h to induce apoptosis [26,27]. This suggests that multiple events, including possibly metabolic events, are likely required for induction of apoptosis by EA. Cells that are under metabolic stress will often undergo autophagy to generate nutrients for survival [28]. Considering that EA may impose metabolic stress on A498 cells, the induction of autophagy in response to EA was determined. The induction of authophagy was examined by three methods, independently, in A498 cells treated with EA. For the first of these series of experiments, A498 cells were treated with 200 nM EA or 0.1% DMSO (control) for approximately 45 h. In addition, cells were treated with rapamycin (500 nM), an agent known to induce autophagy [29], for 20 h. Flow cytometry was performed using the fluorescent probe, Cyto-ID^®^ Green which primarily stains autolysosomes and earlier autophagic compartments. As presented in Figure 3A, flow cytometry analysis clearly revealed increased staining of cells treated with EA (19.8% autophagic) or rapamycin (12.6% autophagic) compared to control (1.9% autophagic) cells suggesting the induction of autophagy. Importantly, under the conditions of the assay, EA appeared to be at least equal to rapamycin in inducing autophagy in A498 cells. In independent experiments, cells treated with EA as above were also examined by fluorescence microscopy after dual staining with Hoechst nuclear stain and Cyto-ID^®^ Green detection reagent. The results displayed in Figure 3B show the increased staining of EA treated cells with Cyto-ID^®^ Green (panel d) compared to control cells treated with vehicle (panel c). Specifically, EA treated cells displayed intensely stained punctate structures representing the spherical vacuoles that accumulate in the perinuclear region, or in foci distributed though out the cytoplasm of cells undergoing autophagy [30,31]. The upper panels of Figure 3B show stained nuclei of control (a) and EA treated cells (b). The use of the Cyto-ID^®^ Green detection reagent enabled detection and quantification of autophagic cells induced by EA, however, to confirm this action of EA at the molecular level, a well accepted indicator of autophagy [32], the conversion of LC3B-I to LC3B-II, was examined by Western blot analysis in EA treated A498 cells. During autophagy LC3-I is converted to LC3-II by lipidation to allow LC3 to be associated with autophagic vesicles. As shown in Figure 3C, Western blot analysis revealed the conversion of LC3B-I to LC3B-II in EA treated A498 cells but not in control cells confirming the presence of autophagic vesicles in EA treated cells. Importantly, the supplementation of culture medium with nonessential amino acids (NEAA), known inhibitors of autophagy [33,34], decreased the level of autophagic vesicles induced by EA (100 nM) in A498 cells (Figure 4A). The fact that there is a decrease in EA-induced autophagic vesicles upon treatment with NEAA, a known inhibitor of autophagy, implies that EA induces autophagy as opposed to causing an accumulation of autophagic vesicles due to reduced turnover or transport to lysosomes [35]. Interestingly, another well known inhibitor of autophagy, 3-methyladenine (3MA), did not inhibit autophagy and was found to be toxic to A498 cells at concentrations above 2.5 mM (data not shown). This is probably due to the dual role that 3MA has in modulating autophagy in which it can actually induce autophagy depending on the temporal patterns of inhibition of class I and III phosphoinositide 3-kinase [36]. In summary, our results demonstrate that EA induces autophagy in A498 cells which can be inhibited by supplementing cell culture media with NEAA.

### Effect of inhibition of autophagy on cell death

Having demonstrated that EA induces autophagy in A498 cells, the question that arises is whether autophagy is a defense mechanism or a cell death mechanism. To answer this question, both cell viability and levels of apoptosis were determined in independent experiments in which A498 cells were treated with and without NEAA (1X) in the presence and absence of 150 nM EA, or with 200 μM VP16 for 46 h. As shown in Figure 4B, the viability of cells treated with EA were similar to that receiving EA plus NEAA as determined by the PrestoBlue^®^ assay. NEAA, alone, had no effect on the cells when compared to control cells receiving vehicle (0.1% DMSO), whereas, cells treated with VP16 lost viability as expected. These results indicated that inhibition of autophagy did not diminish cell death induced by EA. We then examined the levels of apoptosis in A498 cells treated in the same manner as in the viability experiments. The results of these experiments demonstrated that the levels of apoptosis were similar in cells treated with EA compared to those treated with EA plus NEAA indicating that inhibiting autophagy does not affect the level of apoptosis induced by EA (Figure 4C). It is noteworthy that the level of apoptosis induced by EA appears to be much less than that induced by VP16 (Figure 4B) even though the agents reduce cell viability to similar levels (Figure 4A). Taken together, our results suggest that EA-induced autophagy does not appear to be a cell death mechanism, and is likely a defense mechanism that ultimately fails and cells die by a caspase-independent apoptotic cell death and by necrosis (Figures 1B and C).

### Effect of EA on cell cycle

**Figure 5:**
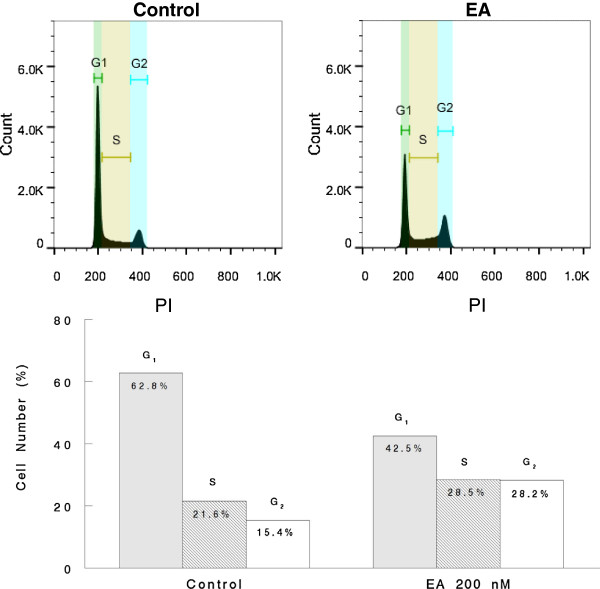
**EA blocks the G**_**2**_**/M transition of the cell cycle.** A498 cells were treated with 200 nM EA or with 0.1% DMSO (control) for 45 h. The cells were then fixed and stained with PI. The PI content of cells was measured by flow cytometry as described under Methods.

In order to gain insight into how EA might regulate cell proliferation in A498 cells, the effect of EA on cell cycle distribution was examined. In these studies, A498 cells were treated with 200 nM EA or with 0.1% DMSO (control) for 45 h. Cells were then stained after fixing and analyzed by flow cytometry as described under Methods. The results from these experiments demonstrated that cells treated with EA accumulated in the G_2_ phase of the cell cycle indicating a block in G_2_/M transition (Figure 5).

### Effect of EA on activation of AKT, ERK, and AMP-activated kinase

**Figure 6:**
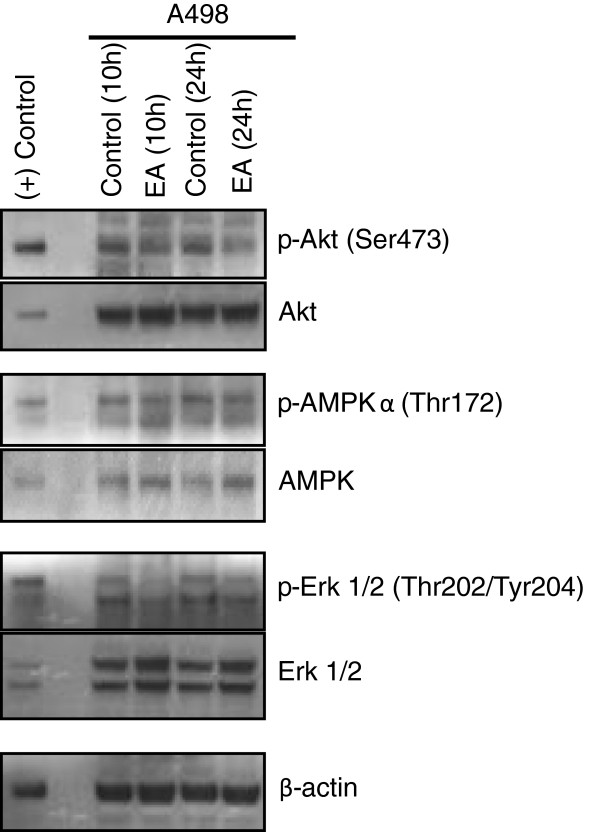
**EA inhibits activation of AKT and ERK kinsases.** A498 cells were cultured with 100 nM EA or with 0.1% DMSO (control) for the indicated times and protein was Isolated. Western blot analysis was performed as described under Methods using antibodies against AKT, ERK, and AMPK and their phosphorylated counterparts. B-actin was probed to control for protein loading. (+) control; Jurkat cell extract.

Because the AKT and ERK signaling pathways drive unrestricted cell proliferation as well as regulate autophagy to obtain nutrients to support rapid growth, they are commonly activated in cancer [37]. Since EA was found to block the cell cycle as well as induce autophagy, it is likely that EA affects these signaling pathways. To examine this possibility, Western blot analysis was performed after treating A498 cells with 100 nM EA or vehicle for increasing times. The results of these experiments revealed reduced levels of phosphorylation of AKT and ERK at both 10 h and 24 h of EA treatment indicating inhibition of both kinases by EA (Figure 6). Inhibition of AKT activation by EA is consistent with its ability to inhibit growth and to induce autophagy. In contrast, activation of ERK is usually associated with induction of autophagy [38]. Activation of AMP-activated protein kinase (AMPK) was also examined since this kinase is a known energy sensor and is activated when ATP levels are low due to cell stress resulting in the induction of autophagy [39]. Interestingly, our results did not reveal activation of AMPK at the time points tested (Figure 6).

In summary, our results demonstrate that EA induces cell death in A498 cells by caspase-independent apoptosis and necrosis while inducing autophagy. Inhibition of autophagy does not diminish cell death by EA suggesting that autophagy is not a cell death mechanism and is likely a survival mechanism which ultimately fails. In addition to inducing cell death, EA arrests cells in G_2_ phase of the cell cycle blocking the G2/M transition. Taken together, our results indicate that cell death by EA occurs by multiple mechanisms which are likely cell context dependent. Because EA can elicit cell death by multiple mechanisms and can inhibit multiple pathways that drive cell proliferation, it has the potential to be an effective chemotherapeutic agent that can bypass chemo-resistance, making it ideal for the treatment of metastatic RCC.

## Discussion

Metastatic RCC is one of the most chemo-resistant cancers for which no curative treatment is available. Hallmarks of this cancer include a highly hypoxic and glycolytic nature and an increased dependency on glucose, all characteristics associated with VHL loss and HIF stabilization which play a central role in the pathogenesis of RCC. However, the limited success of therapeutics targeting the VHL/HIF axis suggests that other molecular alterations also play an important role in the development of RCC. Since pVHL loss and HIFα stabilization are the earliest detectable molecular events in VHL-associated renal tumorigenesis, it is believed that these initial changes trigger other events, both HIF-dependent and independent, resulting in progression to RCC. For example, increased hepatocyte growth factor signaling through c-MET, increased susceptibility to TGF-α/EGF signaling, as well as modifications in extracellular matrix turnover and remodeling are implicated in the pathogenesis of RCC [40]. Clearly, RCC is a complex disease resulting from numerous alterations of genes and pathways that work in concert, indicating that pursuing a single target or pathway will not yield chemotherapeutics with significant efficacy. The best chance for achieving therapeutic efficacy in a disease such as RCC should involve the use of agents that target the multiple pathways which contribute fundamentally to this disease.

Natural products are well known to affect multiple targets and thus have excellent potential as chemotherapeutic agents. The relatively recently identified natural product, englerin (EA), is very unique due to its high selectivity against RCC that is 1000-fold higher than any other cell type [16]. Our results demonstrate that EA induces apoptosis and autophagy in addition to necrosis in A498 RCC cells at nanomolar concentrations. This finding is in contrast to a recent report stating that EA induced necrosis but not apoptosis or autophagy [22]. In this previous study, however, autophagy was most likely inhibited by the supplementation of culture medium with non-essential amino acids (NEAA), a known inhibitor of autophagy [41], and was thus not observed. Our results confirmed that autophagy induced by EA could be inhibited by NEAA. We further showed that inhibition of autophagy by NEAA did not diminish cell death. This finding is supported by the previous study which showed that RCC cells died under conditions which inhibited autophagy with a sensitivity to EA similar to that observed by us and others [16,21]. For instance, in viability assays in the study by Sulzmaier et al. [22], EA was found to have an EC_50_ of 53 nM in the presence of NEAA. In the absence of NEAA, the estimated EC_50_ of EA in A498 cells in our viability assay was 63 nM (Figure 1 and data not shown). Furthermore, the NCI reported LC_50_ for EA in A498 cells, under conditions not inhibiting autophagy, was 79 nM [16]. Though the NCI determined LC_50_ is a somewhat different measure than the EC_50_, determined by us and Sulzmaier et al. [22], in addition to the assays being different, the fact that these values are not very different regardless of whether autophagy is inhibited, indicates that autophagy does not appear to have much of an effect on cell death. Though autophagy can play a pro-death role when prolonged or in certain developmental conditions [42], in most circumstances, autophagic generation of nutrients prevents or delays cell death [43], thus acting as a survival mechanism. It is, in fact, fairly common for cancer cells experiencing stress of different origin to activate autophagy in an attempt to alleviate stress and survive [44]. It is for this reason, that the autophagic machinery has become a therapeutic target. Inhibiting autophagy in tumor cells exposed to cytotoxic agents often results in increased apoptotic cell death [45]. However, we have not observed this in the context of EA-induced apoptosis as the levels of apoptosis were not altered by the inhibition of autophagy by NEAA (Figure 4). It is not entirely clear what role EA-induced autophagy plays in in A498 cells, but it does not appear to represent a cell death mechanism in this context, and most likely is a survival mechanism that ultimately fails.

Although EA induced apoptosis in A498 RCC cells, it did not appear to be a strong inducer of apoptosis as compared to other agents such as VP16 and camptothecin (Figure 4 and data not shown). Interestingly, the report by Sulzmaier et al. [22] concluded that EA did not induce apoptosis in these cells. However, by analyzing not only external exposure of phosphatidyl serine, but also by examining histone-associated DNA fragments, we found that EA did induce some level of apoptosis in A498 cells. The induction of apoptosis by EA was independent of caspase activation suggesting the involvement of non caspase proteases such as cathepsins and calpains [46]. It is likely that the induction of apoptosis by EA is cell context dependent and, thus, may not be induced in all RCC cells, especially, considering that certain cells may have an apoptotic block. In such a case, EA may induce other mechanisms of cell death such as necrosis as observed by Sulzmaier et al. [22]. Our results indicated that EA also induced necrosis as determined by PI staining (Figure 1C). Taken together, our results indicate that EA can induce cell death by multiple mechanisms and that the predominant mechanism will depend on cell context.

In addition to inducing cell death, EA also induced a block in the G2/M transition of the cell cycle in A498 cells. This indicated that EA may likely regulate cell cycle regulatory genes and affect pathways associated with cell proliferation. In fact, our results indicated that EA inhibited activation of both AKT and ERK, members of two pathways commonly activated in cancer, often together [37], and which are associated with unrestricted cellular proliferation and decreased sensitivity to apoptosis-inducing agents [47]. It is known that inhibition of either pathway alone has a negligible effect on tumor growth and survival suggesting that these pathways share downstream targets [48]. The fact that EA can inhibit activation of both pathways suggests that it would be an effective agent in inhibiting tumor growth. This possibility is supported by the findings of a very recent study of EA in athymic mice bearing 786–0 (renal) tumor xenografts [23]. The results of this study demonstrated that EA markedly inhibited tumor growth over a two week period when administered daily at 5 mg/kg intraperitoneally. This study further showed that tumors excised from the EA-treated mice revealed increased inhibitory phosphorylation of the insulin receptor substrate 1 (IRS1) and decreased activity of the PI3/AKT pathway, in line with our *in vitro* results in A498 cells. Based on their *in vitro* results, the authors of this study concluded that EA bound and activated PKCθ to inhibit insulin signaling while, concurrently, activating HSF1, a known inducer of glucose dependence, thus, starving cells of glucose while promoting glucose addiction. However, because the *in vitro* binding studies with EA and PKCθ were indirect without any binding kinetic analyses, it is unclear if PKCθ is a primary target of EA. Furthermore, the experiments demonstrating inhibition of glucose uptake by EA were performed using EA at 10 μM, a concentration of EA approximately 200-fold higher than its IC_50_. It is well established that when cells are starved, the energy sensor, AMP-activated protein kinase, becomes activated by phosphorylation resulting in the induction of autophagy. If EA inhibits glucose uptake, it would be expected to result in a higher ADP/ATP and AMP/ATP ratio and consequent activation of AMPK. Our results, however, did not reveal activation of AMPK by EA at a concentration of 100 nM, a concentration that is highly cytotoxic to A498 cells. Hence, it is possible that the effects of EA on glucose uptake may occur at micro molar concentrations that are much higher than required for cell death (nanomolar) and could represent off-target effects. Moreover, as a natural product, EA would be expected to have multiple targets and most likely has targets in addition to PKCθ. Such targets may include those associated with the ER stress since it is well established that ER stress results in the induction of cell death and autophagy [49]. An example of agent that induces autophagy and cell death by inducing ER stress in RCC includes STF-62247 which targets VHL-deficient RCC [50]. EA may target proteins within the Golgi complex analogous to carminomycin I, a natural product with selective toxicity to VHL-deficient CC-RCC [51].

In conclusion, EA induces cell death via multiple mechanisms and likely has multiple cellular targets. The identification of these targets and pathways affected by this unique agent will be invaluable in understanding the high RCC- selectivity of EA and allow development of highly effective chemotherapeutics for the treatment of metastatic RCC, a highly treatment resistant cancer.

## Abbreviations

EA: Englerin A; RCC: Renal cell carcinoma; HSF1: Heat shock factor 1; VHL: Von Hippel Lindau.

## Competing interests

The authors declare that they have no competing interests.

## Authors’ contributions

AB directed the study, conducted and supervised experiments, and drafted the manuscript. RTW conducted Western blot experiments and well as performed flow cytometry analysis. ALY provided funding and equipment for the project and advised on the project. MBD and ET consulted on project and edited manuscript. In additon, ET provided partial funding for project. All authors have approved the content of the final manuscript.
