# Erratum: renal cancer-selective Englerin A induces multiple mechanisms of cell death and autophagy

**DOI:** 10.1186/s13046-014-0095-4

**Published:** 2014-12-05

**Authors:** Richard T Williams, Alice L Yu, Mitchell B Diccianni, Emmanuel A Theodorakis, Ayse Batova

**Affiliations:** 1 Department of Pediatrics, Hematology/Oncology, University of California, San Diego, CA USA; 2 The Genomics Research Center, Academia Sinica, University of California, Taipei, Taiwan; 3 Stem Cell & Translational Cancer Research Center, Chang Gung Memorial Hospital, Linkou, Taoyuan, Taiwan; 4 Department of Chemistry and Biochemistry, University of California, 9500 Gilman Drive, La Jolla, CA 92093 USA

**Keywords:** ■■■

## Erratum

In the original publication of this manuscript [1], Figure 1 Panel C shows three flow cytometry plots. All values reported in the figure are correct, but a duplicate of the EA plot was mistakenly used for the Vincristine plot. This mistake did not affect our results or discussion as the text refers to the correct experimental values. The corrected Figure 1 is shown below. We apologize for any confusion this may have caused.

**Figure 1:**
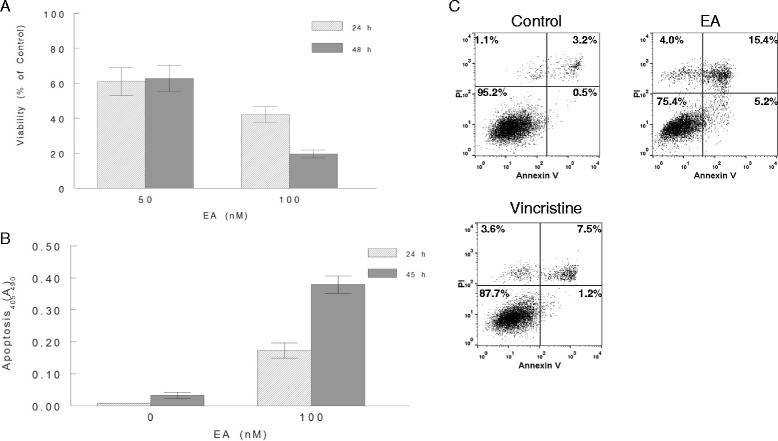
**Induction of cell death by EA in A498 RCC cells.** A498 cells were treated with EA at 50 and 100 nM. Control cells received 0.1% DMSO (vehicle). All conditions were performed in triplicate. Cells were then incubated with additions for 24 or 48 h before measuring viability using the PrestoBlue® assay **(A)**. A498 cells were treated with 100 nM EA or vehicle for 24 and 45 h durations. Apoptosis was determined by measuring cytoplasmic histone-associated-DNA-fragments using the Cell Death Detection ELISAPLUS assay kit **(B)**. A498 cells were treated with 100 nM EA or with 0.1% DMSO (control) for 24 and 46 h. Cells were then trypsinized, washed with ice cold PBS, and stained with Alexa Fluor® 488 annexin V and PI and analyzed by flow cytometry **(C)**.

## References

[1] Williams RT, Yu AL, Diccianni MB, Theodorakis EA, Batova A (2013). Renal cancer-selective Englerin A induces multiple mechanisms of cell death and autophagy. J Exp Clin Cancer Res.

